# Oral Hygiene and Public Education*Read before the Teachers’ Training School of the Kansas City Public Schools, March 12, 1917.

**Published:** 1917-08

**Authors:** Miller Woodson Rice

**Affiliations:** Chief Dental Inspector for the Kansas City Schools


### ORAL HYGIENE AND PUBLIC EDUCATION *

BY MILLER WOODSON RICE, D.D.S., CHIEF DENTAL INSPECTOR FOR THE KANSAS CITY SCHOOLS

I place this occasion upon my calendar as one of the greatest opportunities presented to me, or which might be presented to any member of the dental profession, whereby

* Read before the Teachers' Training School of the Kansas City Public Schools, March 12, 1917.

I may discharge a duty imposed upon me by the dental as well as the medical profession.

The duty of placing the responsibility for the care of children's teeth upon the proper individuals, and I wish to say in this connection that if you are not responsible for this care, you will prove to be the most valuable help the dentist will find in locating and training the ones who are responsible.

The principal duties which have been assigned to the dentists have been those of relieving pain and repairing teeth; we have now come to realize that we are expected to occupy a larger field and it is into this larger field that I wish to direct your attention.

If all the dental schools in this broad land of ours were turning out men to their full capacity and the number of colleges were doubled, we would not then be able to cope with the conditions as they present themselves today, with lost teeth, decayed teeth, dirty teeth, neglected, dirty, diseased gums, and mostly because of the lack of education, care and training, not of dentists, but lack of training of mothers, fathers, sons and daughters. We must bring about a change in this condition of the mouth and when I say we, I mean the dentist, the teacher and the parents.

Let us see what part each of these are to take in preserving healthy conditions of the mouth; the dentist and the teacher know that it is important to cleanse the mouth daily and watch for any defects that might appear in the teeth, and many parents know this, but not all.

For the best welfare of the child all parents should know it, and this knowledge must reach all of them and can be accomplished through the teacher, for those who do not know it seldom visit the dentist and this is going to be more thoroughly accomplished by the younger teachers than by the older ones, for it is hard to break into the curriculum of our older and worthy teachers with new and what seems to be at times unimportant subjects. So I beg of you young teachers to make oral cleanliness on the part of your pupils a part of your work in child training.

Is one of our ward school teachers unduly interested in oral hygiene when she lines up her pupils and looks into their mouths to see if they are clean and sound?

No, she is not living up to her full responsibility as a teacher. She has said to me, "I can see a hole in a tooth, or if they are dirty, by just looking in." The next step is for the dentist, the teacher and the parent to see that daily mouth cleanliness is practiced, which is the hard part. Some one should look into every child's mouth daily and note conditions.

The third step is for the dentist, and he is only the assistant or helper, as he can do the only other thing necessary, make the little repairs that may be needed and help to cleanse the difficult places. This part of the work will be easy for child and dentist if the mouth has been watched daily. A man who has been doing his duty along this line said to me, "My two children are beginning to watch their own mouths." He told me one of them said to him the other day, "I believe I see a hole in one of my teeth, I must go down and see my dentist." She did not say daddy's dentist, but "my dentist." This from a seven-year-old, that's as it should be, that is the relationship that should exist.

No child should escape dental inspection after he begins to erupt teeth. Many need assistance in the process of eruption, such as massaging, gum lancing, consultation, and a visit from the dentist is often needed. After a tooth has made its appearance it should be daily cleansed and watched by the child's attendant until he has reached the age and has been sufficiently trained in daily habits of mouth cleanliness and observation to perform the toilet without assistance; this may be at different ages for different children.

You may wonder how you, as teachers, are to be concerned or helpful in training and caring for children who need this attention as early as twelve months of age or even younger, when you do not have them until they are five and six years of age.

That can be answered in this way, through your interest in community welfare. If I have not been misinformed, the object of parent-teachers associations is for the betterment of conditions that pertain to the schools and the home; and it is through this medium that we hope to reach and benefit the little ones before they reach the school room, enabling them to reach the school room stronger, healthier and more receptive individuals.

It was because of your discovery that the mother needed information along many lines, and that by giving her this information her children might become stronger and better, that you gave up your evenings to meet her in parent-teachers meetings and give her much valuable assistance, and we dentists feel that we are falling far short of our duty to the public we serve if we fail to do our part in the teaching and training of this subject of mouth cleanliness. I assure you no teacher's standing in the community will be sacrificed because she insists on mouth cleanliness, on the other hand she will be the one "whose name leads all the rest."

You may wonder why I place so much stress on oral hygiene, or you may have already answered the question by saying he is a dentist. It is because my experience as a dentist and as an inspector in the public schools has given me a knowledge of conditions that exist, conditions that I know can be prevented by a proper knowledge and care of the mouth, and you as teachers will prove to be the most valuable disseminators of this knowledge. I know not what prompted you to become teachers, but I trust it was because you felt by that means you could be of the greatest service to humanity. No one can serve these little ones from day to day and keep out of their hearts a desire to see them go out with the best equipment possible to battle with the problems of life, and the most fundamental part of this equipment is health.

The importance of this subject will be more fully impressed upon you when you note the significance placed upon it by one of our country's leading surgeons, when he said the greatest per cent. of human ills primarily gain their entrance to the human body through the oral cavity.

I think he placed it as high as ninety per cent. and said that the next great step in medicine must come through dentistry.

If this is true the greater part of this percentage of human ills can be prevented by proper education, care and training of the child in observing oral cleanliness.

I quote you here from the dental inspections of the public schools of last year: number of pupils examined 17,024, number not needing dental attention 1,006, about one in seventeen not needing dental attention, if it were reversed and only one in seventeen needed attention it would be more nearly as it should be.

We dentists can not correct the evil of dental neglect without your cooperation and help. I will say here in defense of any selfish motive on the part of the dentist, that we might as justly accuse you of wishing that people would always remain ignorant so you would always have a job teaching, as for us to wish for present conditions to remain so we would always have dental repairing. If all of the dentist's time was turned toward education and prevention, we would have plenty to do, and I believe the forty dentists who are giving their time without pay to inspect the schools of Kansas City and to making educational talks, and to working in free clinics for the needy, should be recognized and honored as befits true philanthropists.—*Western Dental Journal.*

